# Repellency of *N*,*N*-diethyl-3-methylbenzamide (DEET) during host-seeking behavior of bed bugs (Hemiptera: Cimicidae) in binary choice olfactometer assays

**DOI:** 10.1093/jme/tjae073

**Published:** 2024-06-06

**Authors:** Christopher C Hayes, Coby Schal

**Affiliations:** Department of Entomology and Plant Pathology, North Carolina State University, Raleigh, NC, USA; Department of Entomology and Plant Pathology, North Carolina State University, Raleigh, NC, USA

**Keywords:** bed bug, DEET, repellency, insecticide resistance, behavior

## Abstract

The bed bug (*Cimex lectularius* L.) is one of the most prolific and burdensome indoor pests, and suppression of bed bug populations is a global priority. Understanding bed bug behavior is important to the development of new tactics for their control. Major gaps exist in our understanding of how host cues, insecticide resistance, and exposure modality impact the repellency of formulated products to bed bugs. Here, we validate the use of a binary choice olfactometer for assessing bed bug repellency behaviors using *N*,*N*-diethyl-3-methylbenzamide (DEET) in a dose-dependent manner, while considering the role of host-associated stimuli (with vs. without CO_2_), exposure modality (olfactory vs. olfactory and contact), and resistance status (susceptible vs. resistant) on repellency. We observed that host-seeking insecticide-susceptible bed bugs were repelled only when olfactorily exposed to high concentrations of DEET. However, exposure to DEET by contact repelled insecticide-susceptible bed bugs at 100-fold lower dose of DEET. Further, we demonstrate for the first time that insecticide-resistant bed bugs were significantly more responsive to DEET than susceptible bed bugs. We conclude that the 2-choice olfactometer is an effective tool for assessing the behavioral responses of bed bugs to spatial and contact repellents.

## Introduction

The indoor environment is afflicted by several key arthropod pests, with species that are highly adapted to human-built structures being the most prevalent and persistent. Of these, bed bugs (*Cimex lectularius* L. and *C. hemipterus* F.; Hemiptera: Cimicidae), which have resurged globally over the past two decades, are arguably the most difficult to control, due in large part to the widespread emergence of insecticide resistance (Dang et al. 2017, Lewis et al. 2023). Bed bugs are obligatorily hematophagous but are not known to vector pathogens. Nonetheless, their bites are associated with allergic responses ranging from mild itching to painful infected lesions (Doggett et al. 2012, Hwang et al. 2018). Additionally, bed bugs disseminate in their feces high amounts of histamine, an immune modulator known to cause potentially severe respiratory response in vulnerable individuals (DeVries et al. 2018). The risk of these negative health outcomes increases alongside the burden of infestation, often arising from a combination of small founding propagules and failed eradication efforts (Saenz et al. 2012).

In response, a great deal of recent research has focused on the elucidation of resistance mechanisms in an effort to better inform product development, and the evaluation of new pest control tactics (Romero et al. 2007, Zhu et al. 2010, Lilly et al. 2016, Romero and Anderson 2016, Vander Pan et al. 2019, Dang et al. 2021, Ashbrook et al. 2022). Alongside these efforts, researchers have worked on assessing various natural and synthetic repellents against bed bugs in efforts to develop products that might disrupt host- and aggregation-seeking behaviors (Kumar et al. 1995, Wang et al. 2013, González-Morales et al. 2021b, Krüger et al. 2021, Shi et al. 2021). An example of such use is the impregnation of mattress covers with pyrethroid insecticides—similar in goal to the widespread use of long-lasting insecticide-treated bed nets (LLINS) in vector control programs—to disrupt host-seeking behaviors (Wilson et al. 2020).

In general, LLINs are weaved with large holes throughout to facilitate air circulation, while relying on one or multiple active ingredients (AIs) impregnated into the net to repel and kill host-seeking mosquitoes and sandflies (Karunamoorthi 2011). In fact, it has been shown that LLINs provide ancillary benefits by controlling bed bugs and other domiciliary pests, and therefore communities rely heavily on these benefits in the absence of reliable alternatives (Temu et al. 1999, Malede et al. 2019). Despite this wide reliance, however, little is known about the interactions and overlap between bed bugs and LLINs. We have previously demonstrated that bed bugs readily penetrate commonly deployed LLINs, suggesting that LLINs may fail to repel host- and aggregation-seeking bed bugs, and may impose heavy selection pressure on bed bug populations for insecticide resistance (Hayes and Schal 2022, Hayes and Schal 2024). To date, no formal analysis of the repellency of LLINs to bed bugs exists. Although these products are not designed to target bed bugs, communities rely on them for bed bug control, warranting investigation of these interactions (Malede et al. 2019). In pursuit of this, we adapted and validated a forced-air binary choice olfactometer system to assess the impacts of known and putative repellents on bed bug host-seeking behaviors.

To date, bed bug repellency assays have included the application of repellent compounds or formulated products to exposed host skin in an effort to disrupt bed bug feeding behaviors, barrier applications of products, including insecticides, to disrupt host-seeking or aggregation-seeking, and comparisons of aggregation preferences on treated vs. untreated harborages (Kumar et al. 1995, Moore and Miller 2006, Wang et al. 2013, Liu et al. 2014, Anderson et al. 2018, Vander Pan et al. 2019, Krüger et al. 2021). Many of these studies have relied on DEET (*N*,*N*-diethyl-3-methylbenzamide)—the “gold standard” repellent known to be highly efficacious against both flying and crawling arthropods—as a positive control to validate the assay system (Katz et al. 2008, Bissinger and Roe 2010, Chen-Hussey et al. 2014, Andreazza et al. 2021). It has also been shown that DEET remains highly repellent to insecticide-resistant pests, with some preliminary evidence of this in bed bugs (Leal 2014, Liu et al. 2014, Mengoni and Alzogaray 2018). Given the reliance on and efficacy of DEET, it is surprising that large gaps exist in our understanding of how DEET influences bed bug host-seeking behaviors. For example, we are not aware of any dose–response studies of DEET repellency in bed bugs, or how various host-associated cues and various sensory modalities affect the repellency of DEET in bed bugs.

Therefore, in preparation for a formal assessment of whether bed bugs are repelled away from LLINs and AIs associated with LLINs, we developed and validated a binary choice olfactometer. In this paper, we quantify the dose-dependent olfactory repellent effects of DEET on host-seeking bed bugs. We also compare responses to DEET in susceptible and multi-AI-resistant bed bugs, and the effects of host-associated cues (with vs. without CO_2_) and sensory modalities (olfactory-only vs. olfactory and contact repellency) on bed bug responses to DEET.

## Materials and Methods

### Colony Maintenance and Feeding

Two laboratory-maintained strains of *C. lectularius* were used in this study. The Harold Harlan strain (HH; also known as Ft. Dix) is a commonly used insecticide-susceptible reference strain. It was collected at Fort Dix, New Jersey (USA) in 1973 and has not been challenged with insecticides since collection. The HH strain was maintained on a human host until December 2008, then, in our lab, on defibrinated rabbit blood until July 2021, and on human blood thereafter. The Fuller Mill Road strain (FM) was collected from a residence in High Point, North Carolina (USA) in 2017, and was maintained in our lab on defibrinated rabbit blood until July 2021 and on human blood thereafter. The FM strain is highly resistant to pyrethroids (González-Morales et al. 2022) and moderately resistant to fipronil (González-Morales et al. 2021a).

At the time of use, both strains were maintained at 35%–45% relative humidity, 25 C on a 12:12 (L:D) h cycle and fed weekly on heparinized human blood (supplied by the American Red Cross under IRB #00000288 and protocol #2018-026). We used an artificial feeding system, which has been previously described (Sierras and Schal 2017, Hayes and Schal 2022). The feeding system was housed in a North Carolina State University-approved BSL-2 facility (Biological Use Authorization #2020-09-836). Between feeding sessions, the glass feeders were sanitized with 7.5% sodium hypochlorite and 95% ethanol, and air-dried. Only adult females were used in all assays due to their need to obtain a blood meal between each oviposition cycle and thus high motivation to orient toward a potential host. Within 48 h postfeeding, females were separated from colony jars into groups of 20–30 for a 10–14 d starvation period. Since the females were of unknown ages and likely mated within the colony, at several points during this period, but not within 24 h of the assay, groups of females were moved onto clean folder paper in clean vials (20 mL) to remove all eggs. Individual bed bugs were used for a single bioassay, and then discarded.

### Human Odor Preparation

All human odors used as part of this research were collected from the primary researcher (C. C. H.) following a previously validated and approved SOP for human skin swab collection (NCSU IRB Protocol 14173). In short, no alcohol, spicy, or pungent foods were consumed within at least 24 h of the odor collection. No sooner than 2 h before collection, C. C. H. showered using Cetaphil ultra gentle body wash (Galderma, Fort Worth, TX) and no shampoo. No deodorant was applied, and no strenuous activities performed. Hourly, between 2 and 10 h after showering, C. C. H. cleansed his hands with water (no soap) and once dried, a filter paper (#1, 90 mm diameter, Whatman, Maidstone, United Kingdom) was used to swab a single forearm from wrist to elbow, armpit, and leg from ankle to knee for 15 s. A second filter paper was used to swab the corresponding regions on the other side of his body. Each filter paper was cut into 16 equal pie-shaped pieces (4 cm^2^ each) stored in a glass vial at −20 °C and used within 1 mo.

### DEET Dispenser Preparation

Individual squares (1.5 cm × 1.5 cm; 2.25 cm^2^) or rectangles (1.5 cm × 3.0 cm; 4.5 cm^2^) of #1 Whatman filter papers were placed on an aluminum foil and treated with either acetone (control) or DEET in acetone no more than 24 h prior to use and stored in glass vials (20 mL) at −20°C. Concentrations of DEET (PESTANAL analytical standard, Fisher Scientific, Waltham, MA) ranged from 0.001 to 100 μg/μL. Squares were treated with a single 10-μL application of acetone or DEET and then air-dried for 20 min, while each rectangle was folded and treated with two 5-μL applications (one per side; 2.25 cm^2^) and dried for 10 min after each application. The total dose of DEET is reported throughout, but its concentration per square-cm may be derived by dividing by 2.25 and 4.5, respectively.

### Assay Design

**Fig. 1.**
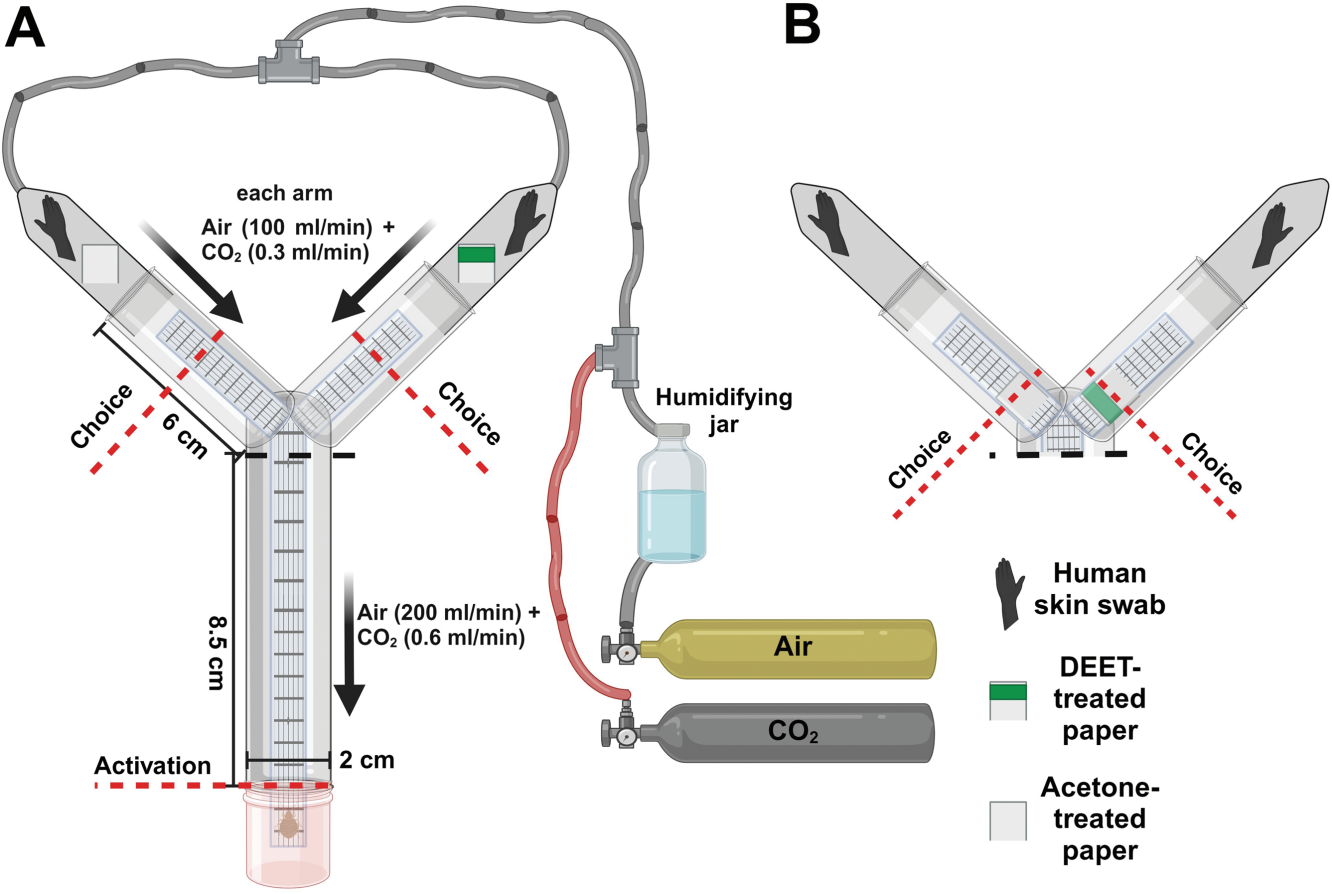
Graphical representation of the binary choice olfactometer used to assess the behaviors of *C. lectularius* to varying doses of DEET. Assays were designed to test olfactory (spatial) repellency independently of contact exposure (A) and olfactory plus contact repellency (B). In both assay designs, host-seeking adult female bed bugs are attracted toward host-associated olfactory cues (human skin volatiles and CO_2_) in humidified air. Behavioral responses to various concentrations of DEET were quantified by measuring percentage activation (bed bugs that entered the olfactometer/total bed bugs assayed); percentage choice (bed bugs that made a choice of either arm of the olfactometer/total bed bugs that activated); arm preference (percentage that chose the treatment vs. control arms); and latency(s) to activation and making a choice, respectively. DEET and human skin swab stimuli were prepared independently and introduced via separate filter papers. Human skin swabs were replaced after each replicate, and walkways were changed after either five replicates or between treatment groups, whichever came first. Created with BioRender.com.

All assays were performed using glass Y-tube olfactometers as previously described (DeVries et al. 2019, Saveer et al. 2021), with minor modifications. Briefly, a vertically oriented 2-cm-diameter glass Y-tube olfactometer with a trunk length of 8.5 cm, arm lengths of 6 cm, and glass odor pots of 5.5 cm inserted into the end of each arm, was connected to a forced-air system (Fig. 1). Medical quality air (Airgas Healthcare, Radnor, PA) was passed through a humidifying jar at 200 mL/min and bifurcated into the olfactometer at the distal end of each odor pot so each arm received 100 mL/min, before rejoining at 200 mL/min in the common arm of the olfactometer. If assays used CO_2_ (Airgas Healthcare, Radnor, PA), it was introduced alongside the already humidified air at 0.6 mL/min (regulated by a low-pressure regulator and a needle valve), which delivered approximately 3,000 ppm of CO_2_. Plankton mesh (Wildco, Yulee, FL) was positioned in the center of the olfactometer and used as a walkway; it was replaced after no more than five replicate assays or for each assayed dose, whichever came first. Individual bed bugs were introduced to the assay via an uncapped releasing jar, and we recorded Activation (moving from the releasing jar into the common arm of the olfactometer), Choice (moving more than halfway up one of the assay arms [Fig. 1A] or crossing onto a DEET- or acetone-treated filter paper walkway [Fig. 1B]), and Preference (selected assay arm).

Prior to their introduction into the assay, each bed bug was placed in a releasing jar for 30 min of acclimatization, and then the releasing jar was attached to a conditioning port for 5–10 min to acclimate to assay airflow conditions. Insects were then introduced to the assay and given 5 min to walk up the common arm of the Y-tube and halfway up one of the assay arms (i.e., make a choice). All assays were run during the scotophase in a dark room at ~25 °C, ~30%–40% RH, with a dimmed red light to facilitate observations.

Two methods of odor introduction were used to assay olfactory-only and olfactory plus contact repellency. First, for olfactory repellency alone, both odor pots contained a 4-cm^2^ pie-shaped piece of a skin swab (fresh swab for each assay) and one odor pot received a DEET-treated square filter paper while the other received a control (acetone-treated) filter paper (Fig. 1A). Second, for olfactory plus contact repellency, both odor pots contained a skin swab, and each walkway in the side arms was affixed with either folded DEET-treated paper or a control (acetone-treated) paper (Fig. 1B). In both cases, bed bugs were challenged to approach host cues (human odor with or without CO_2_) in the presence or absence of DEET.

### Statistical Analysis

All statistical analyses were conducted using SAS Enterprise Guide (v. 8.3, SAS Institute, Cary, NC), with α = 0.05. Analyses of latency to activation and choice were done using Student’s *t*-tests. For comparisons of percentage activation across treatment groups (total number activating by treatment as a percentage of total number assayed), we used a generalized linear model (GLM) followed by Tukey’s HSD on arcsine square-root-transformed data. The percentage of HH bed bugs that made a choice (combined number making a choice at both arms of the olfactometer as the percentage of total number activated) across doses of DEET with and without CO_2_ was analyzed using a chi-square test followed by Holm’s correction for multiple comparisons. The percentage of bed bugs that made a choice across doses of DEET in assays involving both strains was arcsine square-root-transformed and analyzed using one-way ANOVA followed by Tukey’s HSD within each strain, or using a GLM followed by Tukey’s HSD between strains. Dose-dependent percentage preference (one arm as percentage of total that made a choice) was compared via individual chi-square tests based on provided stimuli (CO_2_ vs. no CO_2_) in single-strain assays, and by strain in assays involving both HH and FM strains.

## Results

### Validation of the Olfactometer Assay with Host Cues

**Fig. 2.**
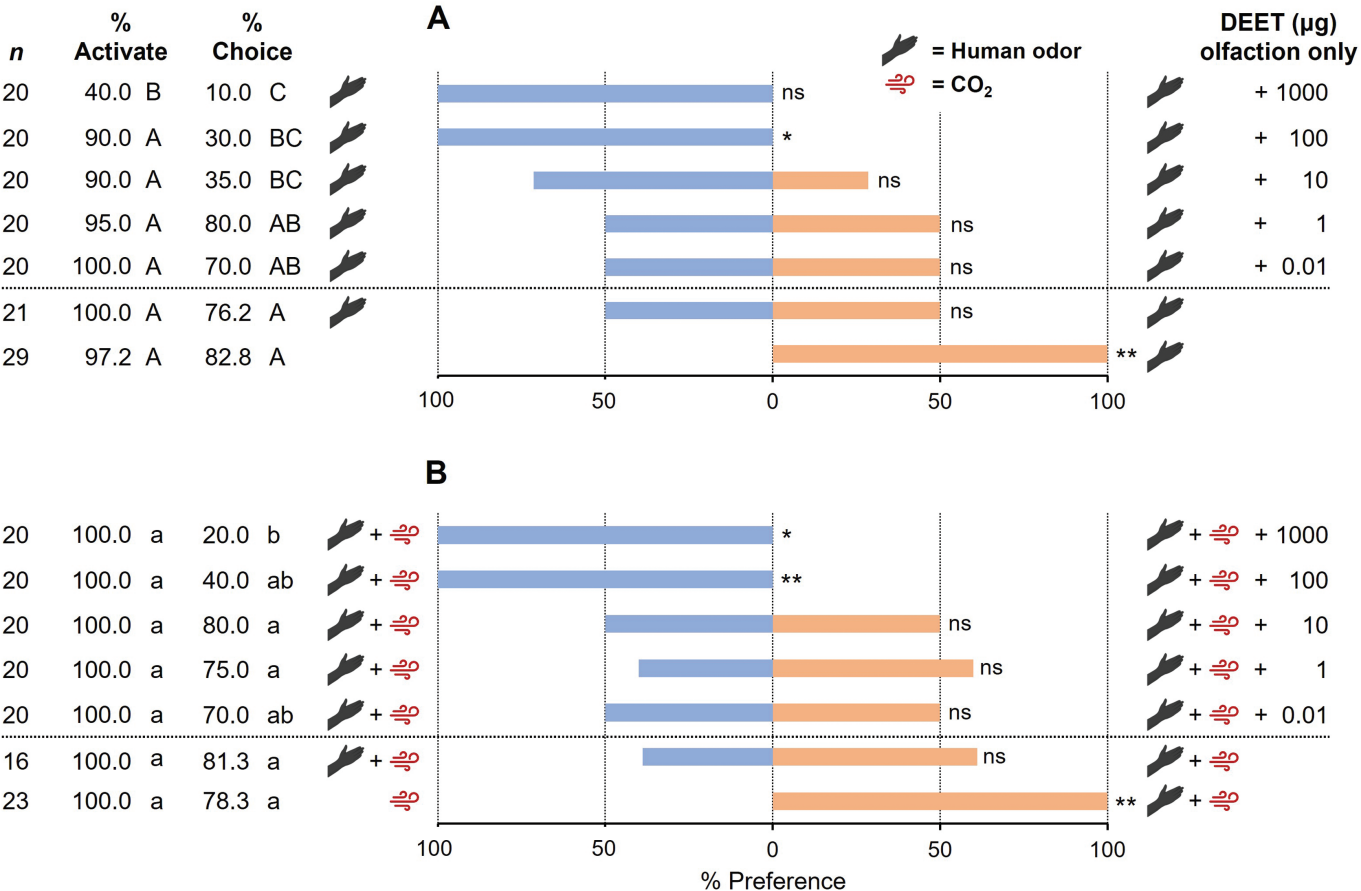
Comparisons of olfactory-mediated behavioral responses of insecticide-susceptible (HH strain) *C. lectularius* to various doses of DEET in the absence (A) or presence (B) of CO_2_. Individual 10–14 d starved females were provided respective host-associated stimuli. The positive control (bottom treatment group in each graph) consisted of host cues (odor and/or CO_2_) at only one arm of the olfactometer, and the olfactometer side-bias control (second treatment group from the bottom in each graph) had identical host cues at both arms. Activation was defined as the female leaving the release vial and entering the olfactometer, and aggregate activation (% Activate) is shown for each treatment group. The percentage of activated bed bugs that chose either arm of the olfactometer is shown as % Choice. The aggregate percentage choosing the subject stimuli, including DEET (right side, orange), and toward the control stimuli (left side, blue) are shown. Percentage Activate was compared within each graph and those that share case-specific letters are not significantly different (One-way ANOVA, Tukey’s HSD). Likewise, % Choice was compared within each graph and those that share case-specific letters are not significantly different (chi-square test, Holm’s correction). Percentage Preference was compared independently within each treatment group (e.g., each dose) by chi-square test, with asterisks representing significant differences denoted by *P < 0.05, **P < 0.01, and ***P < 0.001.

Using the olfaction-only assay (Fig. 1A), we evaluated the effectiveness of the olfactometer with human odor alone and with human odor plus CO_2_ in the absence of DEET. In the positive control, where a human skin swab alone was presented in one arm of the olfactometer and a control filter paper in the other, 97.2% of bed bugs were activated, with 82.8% making a choice, of which 100% chose the human odor arm over the control arm (χ^2^ = 24, *df* = 1, *P* < 0.01; Fig. 2A). The lack of side bias in the olfactometer was confirmed with human odor (no CO_2_) emanating from both arms of the olfactometer, resulting in 100% activation, with 76.2% of the bed bugs making a choice, and 50% choosing each arm of the olfactometer (χ^2^ = 0, *df* = 1, *P* > 0.05; Fig. 2A). Thus, this olfactometer is appropriate for resolving olfactory preferences of bed bugs.

**Fig. 3.**
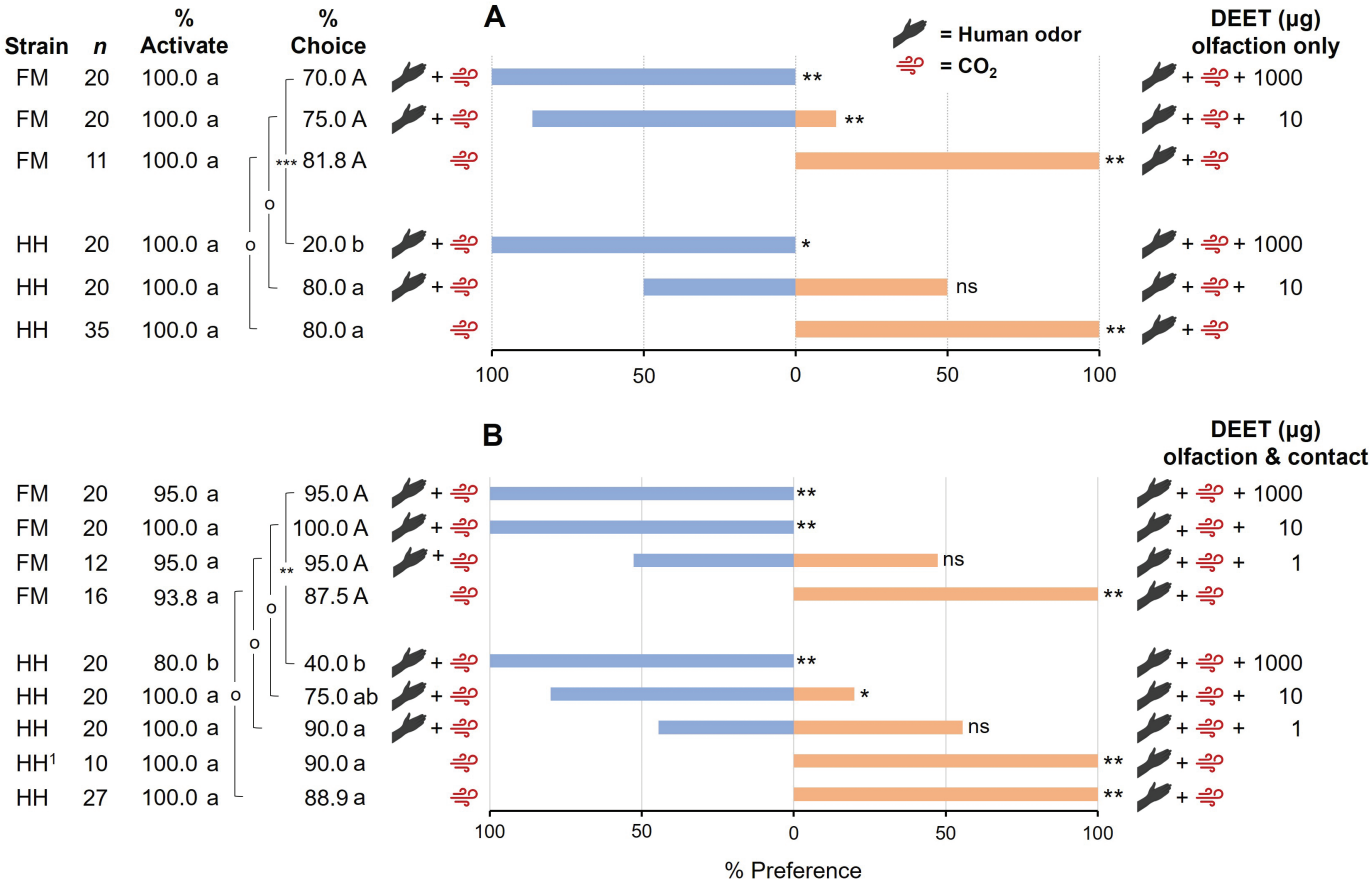
Comparisons of (A) olfaction-mediated and (B) olfaction-plus contact-mediated behavioral responses of insecticide-susceptible (HH strain) and insecticide-resistant (FM strain) *C. lectularius* to various doses of DEET. Individual 10–14 d starved females of both strains were provided host-associated stimuli, namely human odor and/or CO_2_. The positive control (bottom treatment group in each graph) consisted of human odor and CO_2_ at one arm of the olfactometer and CO_2_-only at the other arm. A subset of HH (HH^1^) was run as method validations to ensure that the addition of paper walkways did not significantly alter behavior. The percentages of activated bed bugs that chose either arm of the olfactometer are shown (% choice). The aggregate percentage preference toward the subject stimuli, including DEET (right side, orange), and toward the control stimuli (left side, blue) are shown. Percentages activation, choice, and preference were compared separately based on exposure modality using identical methods. Activation rates were compared across strains, and those that share lowercase letters are not significantly different (GLM, Tukey’s HSD, *P* < 0.05). Percentages making a Choice were compared within strains (One-way ANOVA, Tukey’s HSD) and between strains (GLM, Tukey’s HSD), and those that share case-specific letters are not significantly different. Between strains, comparisons are denoted by brackets, with significance denoted by: o, ns; **P < 0.01; and ***P < 0.001. Preference was compared independently at each dose by chi-square test, with asterisks representing significant differences in preference denoted by *P < 0.05 and **P < 0.01.

Similar results were obtained with the addition of CO_2_ to human odor (Fig. 2B). In both treatment groups 100% of the bed bugs were activated and 78.3% and 81.3% made a choice in the positive control assays (odor plus CO_2_ vs. CO_2_-only) and side-bias assays (both arms with human odor plus CO_2_), respectively. In the positive control treatment group, 100% of the bed bugs preferred the human odor plus CO_2_ arm over the CO_2_-only arm (χ^2^ = 18, *df* = 1, *P* < 0.01) and there was no evidence of side bias when both arms emitted human odor and CO_2_ (χ^2^ = 0.692, *df* = 1, *P* > 0.05). The high resolution and lack of side bias of the olfactometer was further confirmed in all subsequent experiments that compared the olfactory-only preferences (Fig. 3A) and olfactory plus contact preferences (Fig. 3B) of two strains of bed bugs.

### Responses to DEET in the Presence of Host Olfactory Cues

Using the olfaction-only assay (Fig. 1A), we assessed the effects of increasing doses of DEET on bed bug responses in the presence of human odor alone or human odor plus CO_2_. Despite the presence of DEET, an overall high percentage of bed bugs (90.0%–100%) activated in response to human odor alone across all but the highest assayed DEET dose (Fig. 2A). At 1,000 μg of DEET, significantly fewer bed bugs were activated (40.0%) in response to human odor alone (one-way ANOVA, *F* = 9.60, *df* = 7,40, *P* < 0.001; Tukey’s HSD, *df* = 40, *P* < 0.05). However, the addition of CO_2_ to human odor raised bed bug activation to 100% across all doses of DEET (Fig. 2B). Thus, higher quality host cues (host odor plus CO_2_) stimulate bed bugs to overcome the repellency of DEET in search of a blood-host. Conversely, high concentrations of DEET are more effective at repelling bed bugs from lower-quality host cues (host odor alone).

With human odor alone (Fig. 2A), the percentage making a choice declined significantly from 80% at 1 μg DEET to 10% at 1,000 μg DEET (chi-square followed by Holms correction; χ^2^ = 44.54, *df* = 6, *P* < 0.0001). A significant decline in the percentage making a choice was seen at 10 μg (*P* = 0.0304), 100 μg (*P* = 0.0102), and 1,000 μg (*P* < 0.0001) DEET relative to the side-bias control. The addition of CO_2_ to human odor (Fig. 2B) had varying effects but overall increased the percentage of bed bugs that made a choice (χ^2^ = 29.22, *df* = 6, *P* < 0.0001), with a significant decline in the percentage making a choice only at 1,000 μg DEET (*P* = 0.0105). In both sets of assays, no significant effects on preference were evident at ≤10 μg DEET (Fig. 2). In the presence of 100 μg DEET, 100% of the bed bugs preferred the control arm of the olfactometer, regardless of host cue quality (human odor with CO_2_ [χ^2^ = 6, *df *= 1, *P* < 0.05] or without CO_2_ [χ^2^ = 8, *df* = 1, *P* < 0.01]). Likewise, in the presence of 1,000 μg DEET, 100% of the bed bugs preferred the control arm of the olfactometer (no DEET) regardless of provided host cues (human odor with or without CO_2_). However, only 2 of 20 bed bugs responded in the presence of host odor alone (Fig. 2A), whereas 4 of 20 bed bugs responded when CO_2_ was added to host odor (χ^2^ = 4, *df* = 1, *P* < 0.05; Fig. 2B). These findings demonstrate that bed bugs can overcome the presence of a spatial (olfactory) repellent in pursuit of host-emitted cues, except at remarkably high concentrations of the repellent.

### Strain Variation in DEET Olfactory Repellency

To determine if variation in spatial repellency exists between strains, we compared the activation, latency, choice, and preference of the insecticide-susceptible HH strain and the insecticide-resistant FM strain at two doses of DEET, using olfactory-only assays (Fig. 1A). Based on the HH dose–response results with host odor and CO_2_ (Fig. 2B), we used 10 μg DEET, at which HH bed bugs were not repelled, and 1,000 μg DEET, which significantly repelled all bed bugs that made a choice. In both strains, 100% of the bed bugs activated in all treatment groups. Further, a high percentage of bed bugs making a choice was seen in the positive controls of both strains (HH = 80.0%, FM = 81.8%; Fig. 3A). However, whereas in HH bed bugs the percentage that made a choice significantly declined at 1,000 μg DEET to only 20.0% (one-way ANOVA, *F* = 7.24, *df* = 3,15, *P* = 0.0031; Tukey’s HSD, *df* = 15, *P* < 0.05), 70.0% of the FM strain bed bugs made a choice with no significant differences among DEET doses (one-way ANOVA, *F* = 0.68, *df* = 2,9, *P* = 0.5297). Comparison of the two strains based on the percentages of bed bugs making a choice revealed an overall significant model (GLM, *F* = 3.87, *df* = 6,24, *P* = 0.0077), with only dose significantly affecting the percentage making a choice (Tukey’s HSD, *F* = 5.69, *df* = 3, *P *= 0.0043). Neither strain alone (*F* = 3.24, *df* = 1, *P* = 0.0843) nor the interaction of dose and strain (*F* = 2.46, *df* = 2, *P* = 0.1069) significantly affected the percentage making a choice (Fig. 3A). Specifically, no significant difference in the percentage making a choice was seen between the strains at 10 μg DEET (Tukey’s HSD, *t* = -0.54, *df* = 24, *P* = 0.5965), but significantly more of the FM than HH bed bugs made a choice at 1,000 μg DEET (Tukey’s HSD, *t *= -3.95, *df* = 24, *P* = 0.0006; Fig. 3A). The results suggest that high aerial concentrations of DEET are more repellent to the insecticide-resistant FM strain than to HH bed bugs in the presence of host cues.

In both the HH and FM controls, 100% of the bed bugs that made a choice were attracted to human odor and CO_2_ (chi-square test, HH: χ^2^ = 18, *df* = 1, *P *< 0.01; FM: χ^2^ = 9, *df* = 1, *P *< 0.01). The HH bed bugs showed no significant preference at 10 μg DEET, with 50% choosing each arm of the olfactometer (χ^2^ = 0, *df *= 1, *P* > 0.05). However, they were significantly repelled by 1,000 μg DEET, with 100% choosing the host cues-only arm (χ^2^ = 4, *df *= 1, *P* < 0.05; Fig. 3A). In contrast, the FM bed bugs were significantly repelled at both doses (10 μg: χ^2^ = 8.067, *df* = 1, *P* < 0.01; 1,000 μg: χ^2^ = 14, *df* = 1, *P* < 0.01; Fig. 3A). These results, taken alongside our observations that more FM bed bugs make a choice at high DEET concentrations, suggest that the FM strain might be more olfactorily sensitive to DEET than HH bed bugs, resulting in an increased excito-repellent response.

### Strain Variation in Combined Olfaction and Contact Repellency of DEET

To further understand the repellent effects of DEET, we challenged host-seeking bed bugs to orient upwind toward host-associated cues in the presence of DEET volatiles (olfactory repellency) and then traverse a DEET-treated filter paper (contact repellency) using a modified assay design (Fig. 1B). The percentage of bed bugs that activated remained high (≥80%), but the addition of contact exposure reduced activation of HH bed bugs at the highest dose of DEET (GLM, *F* = 2.72, *df* = 9,36, *P* = 0.0157; Fig. 3B). Namely, the dose of DEET significantly affected activation (*F* = 3.46, *df* = 5, *P* = 0.0118), but neither strain (*F* = 0.65, *df* = 1, *P* = 0.4259) nor the interaction of strain and dose (*F* = 2.42, *df* = 3, *P* = 0.0823) had a significant effect. There was significantly lower activation of HH (80.0%) than FM (95.0%) bed bugs at 1,000 μg DEET (Tukey’s HSD, *t* = −3.63, *df* = 35, *P *= 0.0009).

Further, high percentages of both HH and FM bed bugs preferred human odor in the positive control assays (no DEET; Fig. 3B). The pattern within each strain was similar to that with olfactory stimulation with DEET. A significantly lower percentage of HH bed bugs reached the choice point when exposed to 1,000 μg DEET by olfaction and contact (40.0%; one-way ANOVA, *F* = 5.00, *df* = 4,21, *P* = 0.0054; Tukey’s HSD, *P* < 0.05), but FM bed bugs responded equally (87.5%–100%) to these stimuli (one-way ANOVA, *F* = 0.57, *df* = 3,14, *P* = 0.6433; Fig. 3B). Comparison between strains of dose-dependent percentage bed bugs that made a choice revealed an overall significant model (GLM, *F* = 3.86, *df* = 8,35, *P* = 0.0024). All three factors in the model significantly affected the percentage that made a choice: dose (*F* = 2.84, *df* = 4, *P* = 0.0386), strain (*F* = 13.33, *df* = 1, *P* = 0.0008), and the interaction of dose and strain (*F* = 4.22, *df* = 3, *P* = 0.0120). As in the olfaction-only assays, at 1,000 μg DEET, significantly more FM bed bugs made a choice (95.0%) than HH bed bugs (40%; Tukey’s HSD, *t* = 3.33, *df* = 35, *P* = 0.0020).

All bed bugs of both strains preferred to orient to the blend of human odor and CO_2_ over CO_2_ alone in the absence of DEET (χ^2^ = 10, *df* = 1, *P* < 0.01). Also, both strains of bed bugs showed no side preference in the presence of 1 μg DEET (HH: χ^2^ = 0.222, *df* = 1, *P* > 0.05; FM: χ^2^ = 0.053, *df* = 1, *P* > 0.05), suggesting that they did not perceive DEET by olfaction and contact at this concentration. Unlike in the olfaction-only assays, however, HH bed bugs were significantly repelled by 10 μg DEET (80.0%; χ^2^ = 5.4, *df* = 1, *P* < 0.05), and 100% of FM bed bugs oriented away from the DEET arm of the olfactometer. With both strains, 1,000 μg DEET repelled 100% of the bed bugs (HH: χ^2^ = 8, *df* = 1, *P* < 0.01; FM: χ^2^ = 19, *df* = 1, *P* < 0.01; Fig. 3B), as in the olfaction-only assays (Fig. 3A). Overall, the combination of the olfactory (spatial) and contact repellency of DEET resulted in greater repellency of host-seeking bed bugs than olfactory repellency alone.

### Analysis of Latency to Activation and Choice

To further assess the effect of host cue quality, doses of DEET, and strain on bed bug behavior, we compared the latency to activation and latency to making a choice across all treatment groups. The latency results did not follow a clear pattern, so they are presented as Supplementary Information. Considering the quality of host cues (odor only: Fig. 2A, or odor plus CO_2_: Fig. 2B), the latency to activation was not significantly different across the controls and all doses of DEET (Supplementary Table S1). Latency to making a choice was significantly lower with the addition of CO_2_ in the side-bias assays (Student’s *t*-test, *df* = 31, *P* = 0.0033) and at 1 μg of DEET (*t*-test, *df* = 30, *P* = 0.0044), and nearly so at 0.01 μg of DEET (*P* = 0.0818; Supplementary Table S2). In olfaction-only assays, there were no significant differences across the controls, both doses of DEET, or between assayed bed bug strains in latency to activation or choice. In olfaction-plus contact assays, as in other experiments, activation latency did not appear to reveal consistent patterns (Supplementary Table S1). As well, the comparison of choice latencies did not reveal any useful insights, with significantly slower time to making a choice by FM in the positive control (*t*-test, *df* = 37, *P* = 0.0321), and significantly faster time to making a choice by FM at 1 μg DEET (Supplementary Table S2).

## Discussion

To our knowledge, this is the first study to assess the repellency of DEET to bed bugs (1) in dose–response assays of olfactory repellency, (2) in relation to varying the quality of host-associated cues, and (3) by combining the effects of olfactory and contact repellency, and only the second study to consider the potential association between insecticide resistance and repellency of DEET to bed bugs (Vassena et al. 2019). We were able to accomplish this with a binary choice olfactometer that examines upwind orientation to attractive host cues and is highly sensitive to slight differences in the quality of olfactory stimuli that emanate from each of the two arms of the olfactometer. Briefly, we have shown that as the quality of host cues improves (human odor plus CO_2_), so does the propensity of bed bugs to overcome the repellent while orienting toward the host odor. Further, we have shown that the combination of olfactory (spatial) and contact DEET repellency was more effective than olfactory repellency alone. Finally, we observed strain differences in bed bug responses to DEET which may be associated with insecticide resistance—resistant bed bugs were repelled at lower concentrations of DEET than susceptible bed bugs. Our finding that bed bugs are significantly less repelled by DEET in the presence of high-quality human-associated cues highlights the need to conduct repellency assays under more realistic conditions wherein repellents are challenged to disrupt the innate attraction of bed bugs to host or aggregation stimuli.

The use of binary choice olfactometers to elucidate mechanisms of spatial and contact repellency is common in research across insect orders, where either individuals or groups of insects are simultaneously exposed to stimuli, and their orientation is observed (Grieco et al. 2005, Zhu et al. 2015, Brito et al. 2021). Olfactometer-based assays have been pivotal in elucidating chemically mediated insect behaviors and influencing pest management decisions (Grieco et al. 2007, Roberts et al. 2023), but they have been underutilized in bed bug research. Herein, we have demonstrated the utility of binary choice olfactometers to assess the repellency of insecticides and behavior-modifying products to bed bugs, and their sensitivity to resolve changes in repellency in response to various cues, strain variations, and exposure modality.

### Host-Associated Cues and DEET Repellency

Behavioral assays of repellency rely on effective attractants that stimulate directed orientation behaviors, or arrestants that cause the insect to cease activity; the repellent’s effect at disrupting orientation or arrestment can then be quantified (Syed and Leal 2008, Roberts et al. 2023). In bed bug repellency assays, attractants have largely consisted of host cues (e.g., human odor, heat, and CO_2_) or aggregation cues (e.g., bed bug-conditioned harborages), with light often used as an aversive stimulus to drive bed bugs into the dark shelters (González-Morales et al. 2021b, Krüger et al. 2021, Hayes and Schal 2022). Few studies have sought to optimize the quality of attractants or assess the combinatory or synergistic effects of multiple attractants—especially of different sensory modalities—on bed bug orientation, while challenging bed bugs to overcome repellents embedded in the air stream that carries the attractants.

We found that the addition of CO_2_ to human skin odor (i.e., high-quality host cues) significantly increased the number of bed bugs that oriented upwind despite high concentrations of DEET in the attractive air stream (Fig. 2). Although bed bugs ultimately chose the DEET-free arm of the olfactometer, their greater propensity to move toward an attractive host-associated chemical blend, despite high concentrations of DEET, may result in more frequent interactions with repellents in the field, increasing the risk that bed bugs might find gaps in repellent coverage and contact the host. Our results likely underestimate the ability of bed bugs to overcome repellents in the presence of a host, because we did not consider body heat which is known to stimulate orientation, and did not optimize the skin swab or consider human variations in olfactory cues (DeVries et al. 2016). Overall, these findings suggest that the presence of multiple optimized host-associated cues may compromise the efficacy of repellents on host-seeking bed bugs. These observations highlight the importance of using effective attractants representative of field conditions, rather than blank (or solvent) controls in binary choice assays.

### Insecticide Resistance and DEET Repellency

A large body of literature now exists on the relationship between pyrethroid resistance and accompanying changes in sensitivity to repellents. It has been demonstrated that while *kdr*-type mutations that affect activation of the sodium channel provide resistance to pyrethroids, at least two of these mutations had no effect on DEET repellency in *Aedes aegypti* L. (Diptera: Culicidae; Andreazza et al. 2021). However, there is no consensus across insect orders; in some species, insecticide resistance is associated with lower sensitivity to repellents, whereas in other species no clear associations were found (Wagman et al. 2015, Wu and Appel 2018, Fardisi et al. 2019). For example, a field-collected pyrethroid-resistant strain of *C. lectularius* from Argentina was found to have lower sensitivity to DEET than the insecticide-susceptible Harlan (HH) strain (Vassena et al. 2019). In contrast, our assays indicate that the multi-AI-resistant FM strain was repelled at 100-fold lower dose of DEET than HH bed bugs (Fig. 3A), suggesting greater sensitivity to DEET.

The higher repellency at lower DEET concentrations was further confirmed in the combined olfaction and contact assays, where 100% of FM bed bugs made a choice (i.e., oriented upwind and entered either arm of the olfactometer) at 10 µg DEET, yet 100% avoided the DEET-treated walkway at the same dose (Fig. 3B). We posit that the observed excito-repellent behavior, characterized by rapid movement, higher percentage making a choice, and consistent avoidance of DEET, supports the notion of higher DEET sensitivity in the FM strain. Further studies are needed with a range of resistant bed bug populations in which resistance mechanisms and target site mutations are characterized and associated with behavioral changes in sensitivity to repellents. Recent global surveys indicate that few, if any, insecticide-susceptible *C. lectularius* populations exist in the field (Dang et al. 2017, Lewis et al. 2023). Therefore, it is important to understand the far-reaching impacts of widespread insecticide resistance on bed bug behavioral responses to repellents and repellent insecticides.

### Olfactory and Contact Repellency of DEET

Assays of bed bug repellency to DEET have concentrated on contact repellency, rarely considering spatial (olfactory) repellent effects independently. For example, in Petri dish assays, a portion of the substrate is treated with a repellent, and the position of bed bugs is recorded over time (Wang et al. 2013, González-Morales et al. 2021b, Krüger et al. 2021). In this case, both contact and olfactory repellency contribute to assay outcomes, but their respective contributions cannot be disentangled. In contrast, olfactometers that use directional air flow assay olfactory repellency separately from contact exposure, consistent with the recent identification of DEET-sensitive olfactory receptor neurons in *C. lectularius* through single sensillum recordings (Liu et al. 2017). Representing the first known assays of this kind in bed bugs, our results demonstrate that the combined olfactory and contact exposure of bed bugs to DEET significantly elevated the repellency of DEET and required 10- to100-fold less DEET for the expression of repellency in the HH strain relative to olfactory (spatial) repellency alone (Fig. 3B). We suggest that investigating both exposure modalities, olfaction and contact, leads to a deeper understanding of bed bug behavioral interactions with repellents and insecticidal products, as volatilized chemicals are frequently encountered prior to contact with treated surfaces, and novel formulations may amplify or suppress olfactory repellency to enable more effective bed bug management.

## Field Relevance and Conclusion

In summary, we have for the first time assessed the repellency of DEET to the common bed bug *C. lectularius* in a dose-dependent manner, considering the independent modality of olfaction-based exposure, the addition of contact exposure, the impact of differing host-associated stimuli, and the role of insecticide resistance. Despite the extensive use of synthetic repellents, essential oils, and repellent insecticides in bed bug control, large gaps remain in our understanding of bed bug sensitivity to repellents and changes to repellency associated with the volatility of repellents, their mode of application, and insecticide resistance in bed bugs. Certainly, the purpose of these assays was not to suggest the use of DEET as a repellent product in bed bug control, although our work further elucidates the parameters of its use as a standard repellent in bed bug research. Instead, we used DEET as a reference repellent to demonstrate the utility of binary choice olfactometers to assess the repellency of field-applied products to bed bugs.

One such case, as already mentioned, is the widespread use of LLINs to protect sleeping humans from disease vectors, which has relied on the excito-repellent properties of pyrethroid insecticides. The deployment of LLINs in the same environment where bed bugs live (indoors and beds) to protect the same host that be bugs seek (humans) has imposed strong selection pressure on bed bugs, which have become significant pyrethroid-resistant domiciliary pests in malaria-endemic areas (World Health Organization 1968, Busvine and Pal 1969, Kweka et al. 2009, Deku et al. 2021). Treated LLINs have been shown to disrupt mosquito host-seeking behaviors, but their effect on bed bug host-seeking behaviors is still unknown (Parker et al. 2015). Our assays with DEET will guide future work with LLINs to elucidate the potential impacts of vector control on bed bug populations.

## Supplementary Material

tjae073_suppl_Supplementary_Tables_S1-S2
