# Supplementary material for: Repellency of N,N-diethyl-3-methylbenzamide (DEET) during host-seeking behavior of bed bugs (Hemiptera: Cimicidae) in binary choice olfactometer assays
Source: J Med Entomol. 2024 Jun 6;61(4):1016–25. doi: 10.1093/jme/tjae073 (PMC11239792; doi:10.1093/jme/tjae073)
Supplement: tjae073_suppl_Supplementary_Tables_S1-S2 [file tjae073_suppl_supplementary_tables_s1-s2.docx]

| **Treatment** | **Figure 2** | | |  | | | **Figure 3A** | | |  | | **Figure 3B** | | | |  |
| --- | --- | --- | --- | --- | --- | --- | --- | --- | --- | --- | --- | --- | --- | --- | --- | --- |
|  | **No CO_2_** | **CO_2_** | ***P*-value*^c^*** | |  | **HH** | | **FM** | ***P*-value*^c^*** | |  | | **HH** | **FM** | ***P*-value*^c^*** | |
| Positive control^a^ | 87.2 | 57.3 | 0.0763 | |  | 59.4 | | 94.9 | 0.0953 | |  | | 52.8 | 99.5 | 0.0065 | |
| Side-bias control^b^ | 62.0 | 37.6 | 0.1089 | |  | - | | - | - | |  | | 17.8 | - | 0.0023*^d^* | |
| DEET (μg) |  |  |  | |  |  | |  |  | |  | |  |  |  | |
| 0.01 | 65.8 | 53.6 | 0.2864 | |  | - | | - | - | |  | | - | - | - | |
| 1 | 45.6 | 27.8 | 0.1446 | |  | - | | - | - | |  | | 64.7 | 56.3 | 0.3261 | |
| 10 | 84.5 | 48.0 | 0.0719 | |  | 48.0 | | 78.4 | 0.1229 | |  | | 32.2 | 44.2 | 0.1653 | |
| 100 | 67.1 | 58.1 | 0.3297 | |  | - | | - | - | |  | | - | - | - | |
| 1000 | 70.8 | 75.7 | 0.4484 | |  | 75.7 | | 55.3 | 0.2182 | |  | | 46.7 | 35.3 | 0.2815 | |

**Table S1.** Comparison of average latency to activation (s) across strains, doses of DEET, and exposure modalities.

*^a^*Assays run with attractive host cues (human skin swab without (Fig. 2A) or with CO_2_ (Fig. 2B) in one arm and either air only (Fig. 2A) or CO_2_ only (Fig. 2B) in the other arm of the olfactometer.

*^b^*Assays run with attractive host cues in both arms of the olfactometer to assess for potential side-bias of the olfactometer.

*^c^*Comparisons performed via individual Student’s *t*-tests

*^d^*Compared to HH positive control

**Table S2.** Comparison of average latency to making a choice (s) across strains, doses of DEET, and exposure modalities.

| **Treatment** | **Figure 2** | | |  | | **Figure 3A** | | | | |  | **Figure 3B** | | | |  |
| --- | --- | --- | --- | --- | --- | --- | --- | --- | --- | --- | --- | --- | --- | --- | --- | --- |
|  | **No CO_2_** | **CO_2_** | ***P*-value*^c^*** | |  | | **HH** | **FM** | ***P*-value*^c^*** |  | | | **HH** | **FM** | ***P*-value*^c^*** | |
| Positive control^a^ | 224.3 | 190.6 | 0.1116 | |  | | 203.9 | 227.7 | 0.2005 |  | | | 99.5 | 148 | 0.0321 | |
| Side-bias control^b^ | 222.5 | 132.5 | 0.0033 | |  | | - | - | - |  | | | 70.1 | - | 0.1043^ | |
| DEET (μg) |  |  |  | |  | |  |  |  |  | | |  |  |  | |
| 0.01 | 251.5 | 200.9 | 0.0817 | |  | | - | - | - |  | | | - | - | - | |
| 1 | 251.4 | 167.9 | 0.0044 | |  | | - | - | - |  | | | 147.2 | 92.2 | 0.0229 | |
| 10 | 260.3 | 218.0 | 0.1855 | |  | | 218.0 | 195.7 | 0.2652 |  | | | 77.7 | 96.5 | 0.1829 | |
| 100 | 213.3 | 171.9 | 0.2461 | |  | | - | - | - |  | | | - | - | - | |
| 1000 | 255.0 | 189.5 | 0.2989 | |  | | 189.5 | 166.9 | 0.3674 |  | | | 76.0 | 65.1 | 0.3245 | |

*^a^*Assays run with attractive host cues (human skin swabs with/without CO_2_) in one arm and either air (CO_2_ assays) or CO_2_ alone (assays involving both strains) in the other arm.

*^b^*Assays run with attractive host cues in both arms to assess effects of assay design on bed bug latency to choice (s). Namely, sidedness (No CO_2_ vs. CO_2_) and modifying the walking surface (olfaction + contact assays).

*^c^*Comparisons performed via individual t-tests

*^d^*Compared to HH positive control
